# Immunotherapy in Breast Cancer and the Potential Role of Liquid Biopsy

**DOI:** 10.3389/fonc.2022.802579

**Published:** 2022-03-15

**Authors:** Mark Jesus M. Magbanua, Ozge Gumusay, Razelle Kurzrock, Laura J. van ‘t Veer, Hope S. Rugo

**Affiliations:** ^1^Department of Laboratory Medicine, University of California San Francisco, San Francisco, CA, United States; ^2^Helen Diller Family Comprehensive Cancer Center, University of California San Francisco, San Francisco, CA, United States; ^3^Worldwide Innovative Network (WIN) for Personalized Cancer Therapy Consortium, Villejuif, France; ^4^Division of Hematology Oncology, University of California San Francisco, San Francisco, CA, United States

**Keywords:** breast cancer, circulating tumor cells, circulating tumor DNA, liquid biopsy, immunotherapy, biomarkers

## Abstract

Liquid biopsy biomarkers, such as circulating tumor cells (CTCs) and circulating tumor DNA (ctDNA), are noninvasive diagnostics that could complement predictive and prognostic tools currently used in the clinic. Recent trials of immunotherapy have shown promise in improving outcomes in a subset of breast cancer patients. Biomarkers could improve the efficacy of immune checkpoint inhibitors by identifying patients whose cancers are more likely to respond to immunotherapy. In this review, we discuss the current applications of liquid biopsy and emerging technologies for evaluation of immunotherapy response and outcomes in breast cancer. We also provide an overview of the status of immunotherapy in breast cancer.

## 1 Introduction

Predictive and prognostic biomarkers in oncology have played an important role in guiding treatment to improve patient outcomes (1, 2). The recent emergence of liquid biopsy-based biomarkers from blood—e.g., circulating tumor cells (CTCs) (3–5) and circulating tumor DNA (ctDNA) (6–10)—has offered minimally invasive approaches to assess tumor response and survival in early-stage and metastatic breast cancer (11, 12). Blood-based biomarkers have addressed the limitations poised by tissue-based biomarkers because they are more readily accessible than tissue (13). For example, blood markers offer several advantages over tissue assessment because of the ease of serial analysis *via* blood draws and the feasibility of monitoring of recurrence after surgical resection, when no clinically measurable disease is present (i.e., minimal residual disease) (14).

Breast cancer is the most common cancer in women and represents the leading cause of cancer-related deaths in women worldwide (15). A significant unmet need is effective treatment for triple negative breast cancer (TNBC), a particularly aggressive subtype of this disease. TNBC, defined by a lack of estrogen, progesterone, and human epidermal growth factor receptor 2 (HER2) receptors, accounts for 15% to 20% of all breast cancers and typically has a poor prognosis (16). Immunotherapy has revolutionized the management of multiple solid tumors. For TNBC, immune checkpoint inhibitor (ICI) agents targeting programmed cell death protein 1 (PD-1) and programmed death ligand 1 (PD-L1) and combined with chemotherapy have demonstrated significant clinical activity in early-stage and metastatic TNBC, leading to regulatory approval in the U.S. (17–20). However, in the metastatic setting, only a subgroup of patients responds to these agents, and in the early-stage setting it is important to identify those who do not need ICI for optimal outcome. Therefore, it is important to discover predictive biomarkers to identify breast cancer patients who will benefit from immunotherapy.

Currently, the only predictive test for first-line immunotherapy in patients with metastatic TNBC is immunohistochemical (IHC) testing for PD-L1 expression (17, 21, 22). PD-L1 testing of tumor tissue currently lacks standardization to encompass the heterogeneity in the assays, the diversity of antibodies for testing and the assessment platforms (instrumentation), and the thresholds for scoring PD-L1 status. Additionally, there is diversity in the tumor microenvironment compartments that are analyzed (tumor cells, immune cells, or both). In addition to prediction, it is important to detect resistance to immunotherapy and identify biomarkers to monitor breast cancer patients during immunotherapy. Evaluating patient immunotherapy response by imaging presents another challenge, as standard radiologic criteria for assessing response to ICI therapy could miss progression. One of the obstacles is pseudoprogression, described as radiologic enlargement of the tumor mass due to infiltration of leukocytes (23). There is an unmet need to identify sensitive and specific predictive biomarkers to select patients who will benefit from ICI therapy and to avoid unnecessary toxicities and cost. Liquid biopsies could be a potential approach to identify more robust biomarkers associated with ICI. Recent studies have shown that CTCs frequently express PD-L1 and are associated with worse prognosis, and thus, could serve as a useful non-invasive biomarker for real-time assessment of PD-L1 status and estimation of risk of disease relapse and progression (24–30).

**Figure 1:**
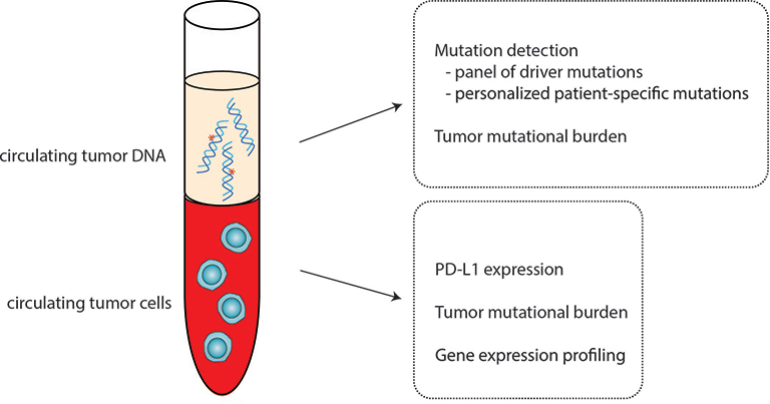
Circulating tumor cells (CTCs) and circulating tumor DNA (ctDNA) as biomarkers for immunotherapy. CTCs and ctDNA can serve as a noninvasive alternative for solid tissue assessment of candidate biomarkers to predict immunotherapy response and outcomes.

In this review, we discuss liquid biopsy applications to guide immunotherapy to treat breast cancer. We highlight the promises and challenges of liquid biopsy biomarkers for breast cancer immunotherapy. Here, we focus our discussion on two liquid biopsy biomarkers, CTCs and ctDNA, and the clinical studies that examined their utility (**Figure 1**).

## 2 Liquid Biopsy Biomarkers: Characteristics and Technology Platforms for Analysis

The most established biomarkers for liquid biopsy assessment include CTCs (31) and ctDNA (6). Over the past decade or more, questions regarding the prognostic and predictive significance of these biomarkers have been actively studied (32–34). Below, we describe CTCs and ctDNA and discuss the detection platforms for each biomarker.

### 2.1 Circulating Tumor Cells

CTCs, defined as rare cells shed by primary tumors into the blood, are hypothesized to be precursors of distant metastases (35). Numerous studies have unequivocally demonstrated the prognostic value of these cells both in early-stage (3, 4) and metastatic breast cancer (5, 36). However, the clinical utility of these cells for guiding treatment to improve patient outcomes has yet to be fully established (37).

The many technologies for the detection and enumeration of CTCs have been reviewed in detail in recent articles (14, 38). To date, the only CTC detection platform to have received clearance from the US Food and Drug Administration (FDA) for enumeration of CTCs in breast cancer is the CellSearch™ system (31). CellSearch™ is a two-step method that involves: (1) immunomagnetic enrichment of cells expressing the epithelial cell adhesion marker (EPCAM), and (2) fluorescence microscopy detection of nucleated cells that are positive for cytokeratin (epithelial marker) and negative for CD45 (leukocyte marker) expression. The detection of 5 or more CTCs per 7.5 mL of blood has been demonstrated to be strongly prognostic for progression-free survival (PFS) and overall survival (OS) in patients with metastatic breast cancer (5, 31, 36). The prognostic value of CTCs in early-stage breast cancer, particularly in the neoadjuvant setting has been recently examined (4). Patients with one or more CTCs identified before neoadjuvant therapy have increased risk of local and distant recurrence as compared to those with no detectable CTCs (4).

Modifications to the standard CellSearch™ protocol for CTC enumeration has allowed for the reliable assessment of PD-L1 expression in CTCs (24, 25, 29, 30, 39). Researchers have added a fluorophore-conjugated antibody to PD-L1 (e.g., B7-H1) to the antibody cocktail (anti-cytokeratin and anti-CD45) for semi-quantitative analysis of PD-L1 expression in CTCs, using cancer cell lines with known PD-L1 expression levels as references. Strati and colleagues used RT-PCR to measure PD-L1 expression in CTC-enriched fractions after immunomagnetic enrichment using CellSearch (25). Others have used filter-based methods to enrich for CTCs prior to immunofluorescence staining to examine PD-L1 expression (26–28).

### 2.2 Circulating Tumor DNA and Cell-Free DNA

ctDNA are short fragments of DNA derived from a primary tumor, metastatic foci and/or circulating tumor cells. ctDNA can be detected in plasma and are present in an admixture of DNA derived mainly from normal blood cells. Collectively, this admixture is known as cell-free DNA (cfDNA). Examination of the size distribution of cfDNA reveals a predominant length of 166 bp with a series of peaks every 10 bp (40). The size and periodicity indicate an association with nucleosomes and suggest that cfDNA is released into circulation *via* apoptosis or necrosis of cells (41). It is unknown whether the mechanisms involved in the release of cfDNA are the same as those of ctDNA (41, 42).

**Figure 2:**
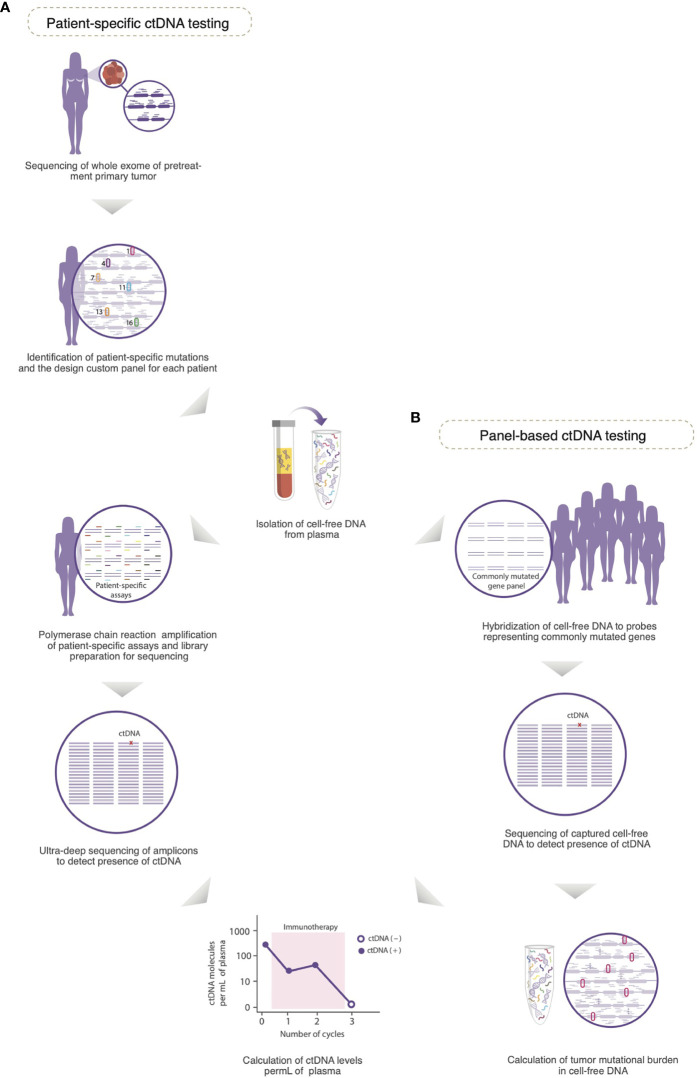
Detection of circulating tumor DNA (ctDNA) in plasma. **(A)** A customized panel containing multiplexed assays is designed to detect patient-specific mutations in cell-free DNA. The personalized panel is created from a list of mutations detected from whole exome sequencing of the untreated primary tumor. Matched germline DNA is also sequenced to exclude non-somatic mutations due to clonal hematopoiesis of indeterminate potential. Amplicons produced by polymerase chain reaction amplification of genomic regions that contain the selected mutations are subjected to ultra-deep sequencing to detect the presence of ctDNA. **(B)** In a panel-based approach, cell-free DNA is hybridized to probes that represent a panel of frequently mutated genes (e.g., *PIK3CA* and *TP53*), and therefore, the mutational profile of the corresponding solid tumor is not required for testing. The captured cell-free DNA molecules are then subjected to next generation sequencing to detect the presence of ctDNA. Because the panel of genes used for testing is consistent across all samples, and includes highly mutated genes, the tumor mutational burden in cell-free DNA can be calculated. In both approaches for testing of ctDNA, serial plasma can be prospectively collected to monitor the levels of ctDNA as a potential biomarker of response to immunotherapy [Modified with permissions from (10)].

Detection of ctDNA can be performed using several methods, including deep next generation sequencing (**Figure 2**) (14). The primary goal of deep sequencing is to detect rare mutated DNA copies shed by tumors (ctDNA) and differentiate them from wildtype copies that are simultaneously released from normal hematopoietic cells undergoing apoptosis. Comparative sequencing studies have shown that specific mutations in ctDNA *vs*. matched primary tumor tissue are generally concordant (43, 44), however, temporal spacing (e.g., timing of sample collection) and tumor heterogeneity could also lead to discrepancies (45). Overall, these data suggest that ctDNA can complement tissue sequencing to find actionable biomarkers. Initial approaches to detection of ctDNA involved digital droplet polymerase chain reaction (ddPCR) (46). However, ddPCR has become less favored (over sequencing) because of its limitations, particularly the restricted number of mutations that can be assessed in one experiment. Sequencing, on the other hand, can interrogate whole genomes, or a panel of genes that include driver mutations frequently observed in cancer, or a personalized list of mutations identified from a patient’s solid tumor (7, 10).

The presence of ctDNA in the blood of patients with early-stage breast cancer is associated with aggressive disease and portends poor clinical outcomes (10, 47). Failure to clear ctDNA during neoadjuvant or adjuvant therapy reflects treatment resistance and increased risk of metastatic recurrence (10, 47).

In the metastatic breast cancer setting, ctDNA testing is becoming a part of routine clinical practice because of the high prevalence of actionable mutations and its potential utility as a surrogate for tumor burden (48). A recently defined clinical use of ctDNA in metastatic breast cancer involves the detection of phosphatidylinositol-4,5-bisphosphate 3-kinase catalytic subunit alpha (*PIK3CA)* mutations, which is already used to guide treatment and is now cleared by the Food and Drug Administration (FDA) (49). Studies are also evaluating the use of ctDNA to detect new mutations during treatment that might represent an early indication of resistance (50), e.g., the emergence of *ESR1* mutations in metastatic breast cancer patients treated with CDK4/6 inhibitor in the PADA-1 Trial (51).

## 3 Immunotherapy in Breast Cancer

Immune checkpoint blockade, which helps the immune system recognize and attack tumor cells, is used to treat various cancers with durable responses compared to most chemotherapy and targeted agents. Inhibiting the PD-L1/PD-1 axis with monoclonal antibodies is a breast cancer treatment strategy that provides cell-mediated antitumor activity. The binding of PD-L1 to its receptor on T cells, PD-1, inhibits adaptive immune responses in the tumor microenvironment, enabling malignant cells to escape immunosurveillance. Immunotherapy drugs approved for the treatment of multiple tumor types include anti-PD-1 (pembrolizumab, nivolumab and cemiplimab), anti-PD-L1 (atezolizumab, durvalumab and avelumab), and the cytotoxic T-lymphocyte antigen 4 (anti-CTLA-4) (ipilimumab and tremelimumab) (52). In the U.S., only pembrolizumab is approved for the treatment of early-stage TNBC in the neoadjuvant setting combined with chemotherapy, followed by adjuvant single agent treatment, and in combination with chemotherapy for PD-L1+ metastatic breast cancer. Atezolizumab in combination with chemotherapy is approved in other countries in PD-L1+ metastatic disease. Multiple ongoing studies are evaluating ICI in all subtypes of breast cancer. Key trials that examined the efficacy of ICI are summarized in **Table 1**.

**Table 1:** Summary of immunotherapy trials in breast cancer.

Trial	Subtype	Ph	ICI arm	Control arm	ORR%	PFS (mo), HR	OS (mo), HR	pCR%
**Metastatic (Single agent ICI)**								
KEYNOTE-086 Coh A (53)	TNBC	II	Pembro	**/**	5.3	2	9	NA
KEYNOTE-086 Coh B (54)	TNBC	II	Pembro	**/**	21.4	2.1	18	NA
KEYNOTE-119 (55)	TNBC	III	Pembro	TPC	9.6 *vs* 10.6	2.1 *vs* 3.3	9.9 *vs* 10.8	NA
NCT01375842 (56)	TNBC	I	Atezo	**/**	10	1.4	8.9	NA
JAVELIN (57)	TNBC	Ib	Avelumab	**/**	5.2	1.5	9.2	NA
JAVELIN (57)	HR+, HER2-	Ib	Avelumab	**/**	2.8	NA	NA	NA
KEYNOTE-28 (58)	HR+, HER2-	Ib	Pembro	**/**	12	l.8	8.6	NA
**Metastatic (ICI+Chemo)**								
IMpassionl30 (ITT) (21, 59)	TNBC	III	Atezo+Nab-pac	PBO+Nab-pac	56.0 *vs* 45.9	7.2 *vs* 5.5 HR=0.80	21.0 *vs* 18.7 HR=0.87	NA
IMpassionl30 (PD-L1 +) (21S;^59^)	TNBC	III	Atezo+Nab-pac	PBO+Nab-pac	58.9 *vs* 42.6	7.5 *vs* 5.0 HR=0.62	25.4 *vs* 17.9 HR=0.67	NA
KEYNOTE-355 (PD-L1 CPS≥ 10) (17, 60)	TNBC	III	Pembro+ Nab-pac /Pac/Gem-Carbo	PBO+ Nab-pac /Pac/Gem-Carbo	53.2 *vs* 39.8	9.7 *vs* 5.6 HR=0.65	23.0 *vs* 16.1 HR=0.73	NA
IMpassionl31 (PD-L1+) (61)	TNBC	III	Atezo+Pac	PBO+Pac	63.4 *vs* 55.4	6.0 *vs* 5.7 HR=0.82	22.1 vs28.3 HR=1.11	NA
NCT03051659 (62)	HR+, HER2-	IIR	Pembro+Eribulin	Eribulin	27.0 *vs* 34.0	4.1 *vs* 4.2 HR=0.80	13.4 *vs* 12.5 HR=0.87	NA
KELLY (63)	HR+, HER2-	II	Pembro+Eribulin	**/**	40.9	6.0	1-year OS 59.1%	NA
**Early-Stage (ICI+NAC)**								
KEYNOTE-522 (20, 64)	TNBC	III	Pembro+Carbo+Pac	PBO+Carbo+Pac	NA	NA	NA	64.8 *vs* 51.2
IMpassion031 (65)	TNBC	III	Atezo+Nab-pac Atezo+AC Postop Atczo xl 1	PBO+Nab-pac PBO-AC Postop observation	NA	NA	NA	57.6 vs 41.1
I-SPY2 (66)	TNBC	II-R	Pembro+Pac	Pac	NA	NA	NA	60 *vs* 20 (est)
I-SPY2 (66)	HR+, HER2-	II-R	Pembro+Pac	Pac	NA	NA	NA	30 *vs* 13 (est)
GeparNuevo (67)	TNBC	II-R	Durvalumab+Nab-pac	PBO+Nab-pac	NA	NA	NA	53.4 vs 44.2
NeoTRIP (68)	TNBC	III	Atezo+Carbo+Nab-pac	Carbo+Nab-pac	NA	NA	NA	43.5 *vs* 40.8

AC, doxorubicin plus cyclophosphamide; Atezo, Atezolizumab; Carbo, carboplatin; Chemo, chemotherapy; Cis, cisplatin; Coh, cohort; Cyclo, cyclophosphamide; DOR, duration of response; Doxo, doxorubucin; est, estimated; gBRCAm, germline BRCA-mutated; Gem, gemcitabine; Gem-Carbo, Gemcitabine-Carboplatin; HER2-, human epidermal growth factor receptor 2 negative; HR, hazard ratio; HR+, hormone receptor positive; ICI, immune checkpoint inhibitor; ITT, intention-to-treat population; mo, months; NA, not available; Nab-pac, nab-paclitaxel; NAC, neoadjuvant chemotherapy; Nivo, Nivolumab; NR, not reached; ORR, objective response rate; OS, overall-survival; Pac, paclitaxel; PBO, placebo; pCR, pathologic complete response rate; PD-L1+, programmed death-ligand 1-positive; Pembro, Pembrolizumab; PFS, progression-free survival; Ph, phase; postop, postoperative; TNBC, triple negative breast cancer; TPC, treatment of physician’s choice; II-R, phase II randomized.

### 3.1 Unresectable Locally Advanced and Metastatic Breast Cancer

Some breast cancers are immunogenic with their tumor microenvironment (TME) enriched with tumor infiltrating lymphocytes (TILs). Increasing evidence suggests that triple negative and HER-2 positive subtypes are often associated with substantial infiltration of immune cells with a prognostic and predictive value (69).

#### 3.1.1 Metastatic TNBC

The primary treatment for metastatic TNBC has been chemotherapy, with a median OS of 12 to 18 months (70). However, growing evidence suggests that immunotherapy is an effective treatment strategy for PD-L1-positive TNBC. Several key factors make TNBC more likely to respond to ICI than other subtypes of breast cancer, including higher levels of TILs, a greater number of nonsynonymous mutations, and higher levels of PD-L1 expression on both tumor and immune cells. High TIL levels are associated with PD-L1 expression on tumor and tumor immune cells (IC), and PD-L1+ tumors with high TILs have better outcomes (54, 71). The emergence of immunotherapy in breast cancer requires robust, sensitive, and specific predictive and prognostic biomarkers for clinical practice. Liquid biopsy could be a valuable tool to provide baseline information on the tumor and to monitor response to ICI therapy.

Although response is higher in TNBC than in hormone receptor positive (HR+) and HER2+ breast cancers, the efficacy of ICI monotherapy, while correlated with tumor and/or immune cell PD-L1 positivity, remains low. The response rates to atezolizumab and pembrolizumab monotherapy were about 5% in patients with pre-treated disease, and ~21% in untreated patients with metastatic TNBC (53, 54). Low response rates with ICI monotherapy led to the investigation of the efficacy of combination therapy with immunotherapy and chemotherapy.

The IMpassion130 trial was the first phase III trial to report positive data with ICI and chemotherapy for breast cancer, investigated the safety and efficacy of nab-paclitaxel +/- atezolizumab as first-line treatment. In this trial, in patients with PD-L1-positive disease, both PFS and OS were significantly improved with the addition of atezolizumab to nab-paclitaxel, by 2.5 (7.5 *vs* 5.0 months, Hazard Ratio (HR) 0.62; p<0.001) and 7.5 (25.4 *vs* 17.9 months, HR 0.67) months, respectively (21, 59). On March 18, 2019, the FDA granted accelerated approval for atezolizumab plus nab-paclitaxel to treat patients with unresectable, locally advanced or metastatic TNBC, whose tumor immune cells express PD-L1 at 1% or higher using the Ventana SP142 assay (72).

In a subsequent trial (IMpassion131), the addition of atezolizumab to paclitaxel in a similar setting failed to improve outcome in patients with PD-L1+ metastatic TNBC (61). Due to the inability to provide confirmatory data for IMpassion130, U.S. approval was withdrawn by the manufacturer (Roche) in August of 2021. The reason for the inconsistency in the results of IMpassion130 and IMpassion131 is not yet understood. A possible explanation for such inconsistency is that patients in the PD-L1-positive control arm in IMpassion131 have a non-stratified pathologic factor that predicts chemotherapy sensitivity (73). Additionally, more patients in IMpassion131 had received prior taxanes than those in IMpassion130, whereas more patients in IMpassion130 had *de novo* metastatic disease than those in IMpassion131 (18, 61). Although exposure to steroids had been considered a potential confounding factor, the use of steroids in the KEYNOTE-355 trial described below makes this unlikely (17, 19).

KEYNOTE-355 randomized patients with metastatic TNBC in the first-line setting to receive pembrolizumab or placebo in combination with physician’s choice of chemotherapy (paclitaxel, nab-paclitaxel, or gemcitabine and carboplatin). The success of this phase III trial resulted in the full regulatory approval of pembrolizumab in combination with chemotherapy in patients with PD-L1-positive TNBC, defined as a combined positive score (CPS) ≥10, and representing about 38% of patients with metastatic TNBC (74). Treatment with pembrolizumab compared to placebo resulted in a statistically significant improvement in PFS (9.7 *vs* 5.6 months, HR 0.65, p=0.0012 and OS (23 *vs* 16.1 months, HR 0.73, p=0.0093), as well as improving objective response rate (ORR, 53.2% *vs* 39.8%) and duration of response (19.3 *vs* 7.3 months) in patients whose tumors expressed PD-L1 (CPS ≥10) (17, 19).

Other chemotherapy agents have been combined with ICI in metastatic TNBC. The ENHANCE phase Ib/II trial evaluated eribulin mesylate (a microtubule-depolymerizing drug) in combination with pembrolizumab in 167 patients with metastatic TNBC, reporting an ORR of 23.4%, with median PFS of 4.1 months and median OS of 16.1 months (62, 75). A small study evaluated the combination of capecitabine with pembrolizumab. Thirty patients were enrolled (16 TNBC, 14 HR+, HER2 negative), reporting a median PFS of 4 months, similar to historic controls with capecitabine alone. Interestingly, the one-year PFS rate was 20.7%, suggesting durable responses in a subset of patients (76).

Preclinical data showed potential synergy with the combination of poly adenosine diphosphate-ribose polymerase (PARP) inhibition and ICI therapy. The phase Ib/II MEDIOLA trial evaluated the safety and efficacy of olaparib with durvalumab in patients with solid tumors, including 34 patients with germline BRCA1/2-mutated HER2 negative metastatic breast cancer. The median PFS was longer in patients who were treatment-naïve than in those with 2 prior lines of chemotherapy (11.7 *vs* 6.5 months; not clearly different than what has been seen with PARP inhibition alone in similar patient populations), and treatment was well tolerated (77, 78). Several other combinations of ICI with PARP inhibitors, AKT inhibitors, MEK inhibitors, antibody drug conjugates, and immunomodulatory drugs, among other drug classes, are under investigation to enhance the host immune response and broaden the subset of patients who could benefit from ICI in the metastatic setting (NCT03167619, NCT04191135). In addition, ICI are being actively studied in various combinations in patients with high-risk HR+ and HER2+ disease.

#### 3.1.2 Tumor Mutational Burden

Tumor mutational burden (TMB) is a promising tool to identify patients with TNBC who could benefit from ICI therapies. In 2020, the FDA granted accelerated approval to pembrolizumab monotherapy in previously treated, unresectable/metastatic solid tumors with high TMB, defined as ≥ 10 mutations per megabase, based upon the results of KEYNOTE-158, which showed an ORR of 29% among 102 patients with 27 tumor types (79). The phase III KEYNOTE-119 study randomized patients with 1-2 lines of prior therapy for metastatic TNBC to receive pembrolizumab *vs* chemotherapy of physician choice, with a primary endpoint of OS. Pembrolizumab did not improve OS, but an intriguing subset analysis demonstrated improved OS in the PD-L1 enriched population (CPS ≥20) (80). A further exploratory analysis suggested a potential positive association between TMB and clinical benefit with pembrolizumab but not with chemotherapy, particularly in patients whose TMB ≥10 mutations per megabase. High TMB is uncommon in breast cancer, representing up to 8% of patients with invasive lobular cancer (81).

#### 3.1.3 Metastatic HR+, HER2 Negative Breast Cancer

HR+, HER2 negative breast cancers have lower TILs and PD-L1 expression levels, so these are traditionally considered immunologically cold tumors (82, 83). However, a minority of patients with cold tumors could have meaningful responses to immunotherapy. The phase Ib KEYNOTE-028 trial evaluated pembrolizumab monotherapy in heavily pretreated patients with HR+, HER2 negative metastatic breast cancer. PD-L1 positivity was defined with a tumor CPS ≥ 1, and among 261 patients, 48 (19.5%) had PD-L1-positive tumors. Of these, 25 patients were enrolled and treated with pembrolizumab. The ORR was 12%, but the median duration of response was 12 months (58). In the phase I JAVELIN trial, 168 patients with pretreated metastatic breast cancer of all subtypes received avelumab monotherapy, including 72 patients (42.9%) with HR+, HER2 metastatic breast cancer, regardless of PD-L1 status. The ORR for the entire cohort was only 3.0% (five patients), including three with TNBC and two with HR+, HER2 negative disease (57). Tolaney et al. conducted a phase II trial evaluating the addition of pembrolizumab to eribulin in HR+, HER2 negative metastatic breast cancer. The addition of pembrolizumab did not improve PFS, ORR, or OS compared to eribulin alone in both the ITT and PD-L1-positive (positivity was defined as modified proportion score ≥1) (84). A multicohort phase Ib study evaluated the efficacy and safety of the combination of pembrolizumab and abemaciclib in patients with HR+, HER2 negative metastatic breast cancer. Early data from 28 patients in the pembrolizumab and abemaciclib arm, all with tumors which had progressed on endocrine therapy, demonstrated an ORR of 29%, with partial response (PR) in 8 patients. Median PFS and OS were 8.9 months and 26.3 months, respectively (84). One arm of this study evaluating the safety and preliminary anti-tumor activity of abemaciclib plus pembrolizumab and anastrozole demonstrated a numerically higher rate of transaminase elevations and pneumonitis which were considered immunotherapy related toxicity (84). Ongoing trials will illuminate the role of immunotherapy in HR+, HER2 negative disease in the coming years (NCT03147287, NCT04895358).

#### 3.1.4 Metastatic HER2 Positive Breast Cancer

Higher levels of TIL infiltration and PD-L1 expression have generated interest in the possible value of ICI in the treatment of HER2+ breast cancer (85). In the phase Ib/II PANACEA trial, pembrolizumab plus trastuzumab had modest efficacy; 6 of 40 (15%) patients with PD-L1-positive disease progressing on prior anti-HER2 targeted therapy achieved an objective response whereas no patients responded in the PD-L1-negative cohort (86). In the KATE2 phase II randomized trial, 202 patients with previously treated HER2+ metastatic breast cancer were randomized to receive atezolizumab or placebo with trastuzumab emtansine. The trial met its futility endpoint due to toxicity in the combination arm, and PFS was not improved with the addition of atezolizumab (87). There are ongoing trials evaluating ICI agents in patients with metastatic HER2+ breast cancer (NCT03199885, NCT02849496).

### 3.2 Early-Stage Breast Cancer

In the early-stage setting, neoadjuvant chemotherapy has resulted in significant improvements in the management of stage II and III TNBC and HER2+ breast cancer (88). Improvements in pathologic complete response (pCR) are associated with excellent outcome, and post-surgical treatment for patients without pCR has reduced the likelihood of recurrence in this high-risk patient population (89, 90). Use of ICI in early TNBC was driven by encouraging results in the phase II I-SPY2 trial (66), and the association of PD-L1 positivity and TILs with pCR (91).

Two phase III trials have evaluated the addition of ICI to neoadjuvant chemotherapy, then continued post-surgery. The largest trial is KEYNOTE-522, leading to the first regulatory approval of a checkpoint inhibitor in early-stage breast cancer. This phase III trial randomized 1174 patients with stage II or III breast cancer in a 2 to 1 ratio to receive neoadjuvant pembrolizumab or placebo in combination with paclitaxel/carboplatin followed by anthracycline/cyclophosphamide. Following surgery, patients continued with blinded pembrolizumab or placebo to complete one year of therapy. In the first 602 patients, the addition of pembrolizumab significantly improved pCR (from 51.2 to 64.8%, P=0.00055), independent of PD-L1 positivity. The trial was designed with dual primary endpoints, including both pCR and event free survival (EFS). At the 4^th^ interim analysis, the addition of pembrolizumab improved EFS at three years (from 76.8% to 84.5%) (64, 80). Interestingly, EFS was improved with pembrolizumab, in the patients who did not achieve a pCR, whereas patients with a pCR had excellent outcome regardless of the post-neoadjuvant treatment arm. Immune-related adverse events (irAE) increased, with 3 deaths attributed to study therapy. Based on this data, the FDA approved pembrolizumab for high-risk, early-stage TNBC in combination with chemotherapy as neoadjuvant treatment, continued as a single agent as adjuvant treatment after surgery on July 26, 2021 (92, 93).

The second phase III trial, IMpassion031, randomized 333 patients with stage II or III TNBC to receive atezolizumab or placebo with neoadjuvant nab-paclitaxel followed by doxorubicin/cyclophosphamide. Following surgery, atezolizumab was continued in a non-blinded manner to complete one year of therapy. The addition of atezolizumab was associated with a significant increase in pCR (from 41.1% to 57.6%, p=0.0044) regardless of PD-L1 expression (65).

The phase II GeparNuevo trial evaluated the efficacy of durvalumab in combination with neoadjuvant chemotherapy in 174 patients. Although pCR was not significantly improved with the addition of durvalumab, invasive disease-free survival (iDFS), distant disease-free survival (DDFS) and OS were improved with long-term follow-up (67, 94). These results, although not definitive, have brought into question the optimal duration of ICI in the treatment of early-stage disease. Lastly, the NeoTRIP phase III trial evaluated the addition of atezolizumab to a non-anthracycline, nab-paclitaxel and carboplatin backbone in 280 patients did not show improvement in pCR although the primary endpoint is EFS, which is still pending (68).

Ongoing trials are evaluating the effectiveness of ICI in the adjuvant and post-neoadjuvant setting. NSABP B-59/GeparDouze is an ongoing phase III trial evaluating neoadjuvant administration of atezolizumab with neoadjuvant chemotherapy followed by adjuvant atezolizumab in patients with high-risk TNBC (NCT03281954). IMpassion030 is a phase III trial investigating the efficacy of and safety of atezolizumab in combination with standard anthracycline/taxane adjuvant chemotherapy in patients with early-stage TNBC (NCT03498716). The primary endpoint is iDFS. SWOG S1418/BR006 (NCT02954874) is a phase III trial that randomizes patients with TNBC and ± 1cm residual invasive breast cancer and/or positive lymph nodes after neoadjuvant chemotherapy to receive standard of care or pembrolizumab 1 year after surgery. The I-SPY2 trial, an adaptive, randomized phase II trial in the neoadjuvant setting also has immunotherapy arms including cemiplimab, cemiplimab plus REGN3767, triaciclibdostarlimab, dostarlimab plus oral paclitaxel/encequidar, and dostarlimab plus oral paclitaxel/encequidar±carboplatin (NCT01042379). Complementary approaches to enhance immunogenicity, including the addition of targeted therapies, novel agents, and induction therapies, have become the recent focus of various clinical trials in breast cancer.

Immune checkpoint blockade can lead to activation of autoreactive T cells, resulting in various irAEs. Although any organ system can be affected, irAEs most commonly involve the gastrointestinal tract, endocrine glands, skin and liver (95). Neurotoxicity, cardiotoxicity and pulmonary toxicity are relatively rare but can be fatal. Whether these adverse events are associated with the efficacy of immune checkpoint blockade remains controversial. The occurrence of irAEs is not required to obtain a benefit from ICI (96). However, specific adverse events may be related with treatment efficacy. For example, several studies including patients with melanoma have demonstrated an association between vitiligo and beneficial clinical outcomes (97, 98). Liquid biopsy biomarkers could also be developed to identify patients who are likely to experience irAEs.

## 4 Predictive and Prognostic Value of CTCs in Immunotherapy in Breast Cancer

Although the presence of PD-L1 has been shown to have good predictive value for ICI efficacy in metastatic TNBC, many challenges persist. First, PD-L1 immunohistochemistry assessment is not always possible due to the lack of available tissue or a low percentage of tumor cells in the tissue sample. Secondly, some patients with PD-L1-positive tumors may not respond to ICI, demonstrating the complexity and our incomplete understanding of the immunopathology of cancer (92). Some challenges are the heterogeneity and dynamic changes of PD-L1 expression in the tumor microenvironment, PD-L1 expression may vary between primary tumors and metastases, and in breast cancer immunotherapy trials, there were multiple assays for each antibody, multiple scoring systems, and different cut-offs to define PD-L1 positivity (99). To address these complexities, PD-L1 expression on the CTCs of metastatic breast cancer patients is actively under investigation as a predictive biomarker for PD-1/PD-L1 inhibition, potentially complementing or replacing PD-L1 detection on tumor cells and/or TILs in tumor tissue.

Liquid biopsy can identify potentially predictive biomarkers for various solid tumors. This approach is appealing since it is minimally invasive, cost-effective, and rapidly provides information to the clinician to guide therapeutic decision-making strategies (100). Liquid biopsy can be repeated longitudinally over the course of the disease, providing follow-up data for the patient during ICI therapy and beyond, and could help detect resistance mechanisms. CTCs can be isolated and analyzed using approaches designed for solid tissue biopsy, and therefore, could be a dynamic and promising strategy. Immune checkpoint proteins can be influenced by multiple factors, including micro-environmental, inflammatory, and therapeutic factors (27). CTCs may be derived from more than one tumor site and give a better systemic representation of PD-L1 expression than the evaluation of localized cells in tissue samples. There are some questions about the evaluation of PD-L1 expression on CTCs. The first is whether PD-L1 is expressed on all CTCs or only in a subpopulation of CTCs. The second is whether there is any discordance in PD-L1 expression between CTCs and the matched tissue biopsies. Lastly, does the prognosis and the predictive response to immunotherapy correlate with PD-L1 expression on CTCs at baseline or during the follow-up of treated patients (101)?

The evaluation of PD-L1 expression on CTCs has been reported in different solid tumor types including breast, lung, head and neck, colon, bladder and prostatic carcinoma (101). Previous studies evaluating the predictive and prognostic value of PD-L1-positive CTCs in patients treated with ICI have revealed provocative results. Nicolazzo et al. monitored CTCs in non-small cell lung cancer (NSCLC) during nivolumab treatment to investigate the association of PD-L1-positive CTCs with response to ICI therapy. At baseline, 20/24 (83%) patients were positive for CTCs with a very high prevalence of PD-L1 expression (100%). At 6 months of treatment, patients with PD-L1-negative CTCs all showed clinical benefit, while patients with PD-L1-positive CTCs experienced disease progression (39). Strati et al. including patients with head and neck cancer (HNC) reported that patients with CTCs overexpressing PD-L1 at the end of treatment had shorter PFS and OS (25). Similar findings were found by Guibert et al. in NSCLC. In this study, 96 patients with metastatic NSCLC receiving chemotherapy followed by ICI were included. PD-L1 was more highly expressed on CTCs (83%) than in matched tissue samples (41%). They found that patients with PD-L1-positive CTCs had lower response rates to nivolumab than those with PD-L1-negative CTCs. All patients who experienced disease progression had detectable PD-L1-positive CTCs (26). In another study including 71 patients with metastatic NSCLC, PD-L1 expression on CTCs and matched tissue biopsies were well correlated (27). Kulasinghe et al. isolated CTCs in 23 patients with HNC and in 33 patients with NSCLC. Positive PD-L1 expression was detected in 6/11 (54.4%) HNC samples and 11/17 (64.7%) NSCLC samples, respectively. PD-L1-positive CTC patients with HNC had shorter PFS while no significant difference in PFS was observed in the NSCLC cohort when stratified by PD-L1 CTC status (28). Another prospective study in 54 patients with advanced NSCLC evaluated the correlation with clinicopathological variables and prognostic value of PD-L1-positive CTC. CTCs and PD-L1-positive CTCs were detected in 43.4% and 9.4% of patients with NSCLC. The concordance of PD-L1 expression between tumor tissue and CTCs was low (54%). This study suggested that the presence of PD-L1-positive CTCs was associated with poor prognosis in patients with advanced NSCLC (30). Taken together, these studies demonstrate the feasibility of PD-L1 testing in CTCs and provide evidence of the predictive and prognostic value of CTCs expressing PD-L1.

**Figure 3:**
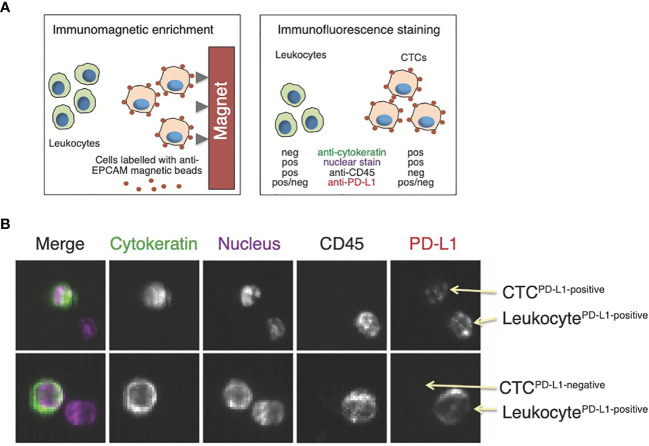
Assessment of PD-L1 expression in circulating tumor cells (CTCs). **(A)** CellSearch is a semi-automated two-step system used for CTC detection. First, monoclonal antibodies against the epithelial cell adhesion marker (EPCAM)-conjugated to iron beads are added to the blood sample. Magnetic capture allows for the enrichment of tumor cells expressing EPCAM. This is followed by immunofluorescence staining to distinguish CTCs from leukocytes and to detect PD-L1 expression; **(B)** Examples of images from the CellSearch gallery to identify CTCs expressing PD-L1. Modified with permission from (24).

Studies on CTCs in breast cancer patients receiving immunotherapy are summarized in **Table 2**. Mazel et al. evaluated the frequency of PD-L1 expression in patients with HR+, HER2 negative breast cancer (24). PD-L1 expression on CTCs was evaluated in breast carcinoma patients using the EPCAM dependent CellSearch method as well as the B7-H1 PD-L1 monoclonal antibody (**Figure 3**). This study included 16 metastatic breast carcinomas with PD-L1-positive CTCs detected in 11 of 16 patients (68.8%), although the fraction of PD-L1-positive CTCs varied from 0.2 to 100% in individual patients. This study was the first report demonstrating the expression of PD-L1 on CTCs (24). The detection of CTCs expressing PD-L1 could be predictive of response to anti-PD-L1 therapy, and patients with a high percentage of PD-L1-positive CTCs could be potential candidates for anti-PD-L1 therapy. In a follow-up prospective study in 72 patients with metastatic breast cancer, CTCs and PD-L1-positive CTCs were detected in 57 (79.2%) and 26 (36.1%) patients before initiation of treatment (29). There was no statistically significant correlation between PD-L1 expression in tumors *vs*. that of CTCs. PD-L1-positive CTCs were significantly associated with PFS while tissue PD-L1 expression was not. Patients with metastatic breast cancer harboring PD-L1-positive CTCs had shorter PFS; however, this finding was not confirmed in multivariable analysis. Further studies are needed to investigate the predictive role of PD-L1 expression in tumor tissue and CTCs during ICI therapy (29).

**Table 2:** Studies on CTC in breast cancer patients receiving immunotherapy.

Setting	Liquid Biopsy Technology	Endpoints	Sample	Results	Reference
Metastatic	CellSearch System (Veridex-LLC, Warren, NJ)	To evaluate the clinicopathological correlations and prognostic value of PD-L1 positive CTCs	72	Baseline CTCs and PD-L1-positive CTCs were detected in 57 (79.2%) and 26 (36.1%) patients. PD-L1 positive CTCs was significantly associated with PFS while tissue PD-L1 expression was not.	(29)
Metastatic	Triple immunofluorescence staining	To evaluate the incidence and clinical relevance of CTC expressing CD47 and/or PD-L1	98	The detection of high CD47 and/or PD-L1 expression on CTC is associated with shorter PFS (5.8 *vs* 13.3 months, p=0.010), whereas the detection of PD-L1 high CTC only was correlated with reduced OS (23.8 *vs* 35.7 months, p=0.043).	(102)
Metastatic and early-stage BC	Maintrac® method	Real-time liquid biopsy to determine PD-Ll and PD-L2 expression	Total=l28 BC=72	PD-L1 expressing CTC were detected in 94.5% of BC patients. Patients with non-metastatic BC had significantly more PD-L1-positive CTC than patients without metastasis (median 75% versus 61.1%; p <0.05).	(103)
HR+, HER-2 negative metastatic BC	CellSearch System (Veridex-LLC, Warren, NJ)	The frequency of PD-Ll expression	16	PD-L1 expressing CTC were detected in 11/16 patients with BC (68.8%) at baseline. The proportion of PD-L1-positive CTC varied from 0.2 to 100% in individual patients.	(24)
HER2 positive, early-stage BC (node-positive)	CellSearch System (Veridex-LLC, Warren, NJ)	Enumerating CTC for monitoring the response to a preventive HER/neu E75 peptide vaccine	16	CTC were detected in 14 of 16 (88%) patients. A significant reduction in HER2/neu- expressing CTC was observed in patients vaccinated with HER2/neu protein derived immunogenic peptide.	(104)

BC, breast cancer; CETC, circulating epithelial tumor cells; CTC, circulating tumor cells; HER2, human epidermal growth factor receptor 2; HR+, hormone receptor positive; PD-L1, programmed death ligand 1; PD-L2, programmed cell death ligand 2.

Schott et al. examined PD-L1 and PD-L2 expression in CTCs of 72 patients with breast cancer (103). CTCs expressing PD-L1 were found in 94.5% of patients using the Maintrac^®^ method. In patients expressing PD-L1 and PD-L2, the proportion of PD-L1-positive CTCs was significantly higher than that of PD-L2-positive CTCs (54.6% versus 28.7%; p <0.001). Furthermore, PD-L1-positive CTCs were detected in patients without metastatic disease, a finding that could extend the use of PD-L1 testing of CTCs in the early-stage setting. Additionally, patients with metastatic breast cancer had significantly more PD-L1-positive CTCs as compared to patients without metastasis (median 75% *vs* 61%; p<0.05). Dynamic monitoring of PD-L1 expression on CTCs during ICI therapy revealed that the number of CTCs and the percentage of the PD-L1-positive CTCs were reduced in patients that responded to ICI therapy. After discontinuing the ICI agent, the percentage of PD-L1-positive CTCs continuously increased. These findings demonstrated that the number of PD-L1-positive CTCs could be prognostic and correlates with tumor aggressiveness, as well as the potential response to immunotherapy (103).

CD47 is a key immune checkpoint which is highly expressed on a variety of cancer cells, making tumor cell resistant to host immune surveillance. Cell surface CD47 is a ligand for signal regulatory protein-α (SIRPα), a protein expressed on macrophages and dendritic cells, allowing cancer cells to send inhibitory signals to macrophages and impede phagocytosis and immune response (105, 106). Agelaki et al. evaluated the incidence and clinical relevance of CTCs expressing CD47 and/or PD-L1 in patients with metastatic breast cancer. Cytokeratin positive CTCs were detected in 22 of 98 patients (22.4%) with metastatic breast cancer. High CD47 and PD-L1 expression was identified in 41.9% and 11.6% of CTCs, respectively, with 9.1% of CTCs expressing high levels of both markers. High CD47 and/or high PD-L1 CTCs were associated with disease progression (27.8% *vs* 5.6%; p=0.005) and shorter PFS (5.8 *vs* 13.3 months; p=0.010), whereas the detection of high PD-L1 CTCs only was correlated with reduced OS (23.8 *vs* 35.7 months, p=0.043). The study showed that high CD47 and/or high PD-L1 CTCs were associated with increased risk of relapse and high PD-L1 CTCs were associated with high risk of death (HR 4.8; p=0.011). Patients with these CTC biomarker-positive populations could benefit from anti-CD47 and anti-PD-L1 immunotherapy strategies (102).

Quantification of CTCs to monitor response to the HER2/neu E75 peptide vaccine was evaluated in 16 patients with HER2+ breast cancer. Patients with node positive breast cancer were vaccinated monthly for six months after completion of standard therapy including surgery, chemotherapy, and radiotherapy. CTCs were detected in 14 of 16 (88%) patients at baseline. A significant reduction in HER2/neu- expressing CTCs was observed over the course of vaccination (104). This small pilot study suggested a potential role of CTCs enumeration in assessing response to vaccine-based therapy; however, these results were not validated in larger studies.

The persistence of PD-L1-positive CTCs in patients treated with ICI therapy in various cancer types has been associated with worse prognosis (39). However, there is no prospective data, and there are technical issues associated with the detection of CTCs: CTCs are rare and various methods might enrich CTCs populations differently, which could affect the PD-L1 assessment. However, liquid biopsy is a promising technique and a feasible strategy for dynamic assessment and sequential monitoring of PD-L1 expression in patients with breast cancer receiving ICI therapy. Given the small number of studies in patients with breast cancer, further studies are needed to understand the role of PD-L1 expression on CTCs during immunotherapy and to determine the relationship between the expression of PD-L1, CTCs, and tumor tissue.

## 5 Predictive and Prognostic Value of ctDNA in Immunotherapy In Breast Cancer

ctDNA detectable in blood has been demonstrated to reflect the mutational signatures of a primary tumor. ctDNA is emerging as a potential noninvasive biomarker to detect preclinical metastases and predict relapse following treatment for early-stage disease. ctDNA provides noninvasive access to cancer-specific somatic mutations and could be a technique used to identify specific mutations that are linked with therapeutic response (107, 108). However, ctDNA has not been used clinically for breast cancer patients treated with ICI.

Baseline ctDNA concentration and genomic instability number have been shown to predict response to ICI, and ctDNA monitoring could become a valuable tool for therapy guidance in the future. Genetic analysis of ctDNA is feasible and thus permits the assessment of TMB, which could be a novel biomarker for cancer immunotherapy. Araujo et al. demonstrated that high TMB could predict ICI efficacy in patients with metastatic breast cancer. Among the 16 patients with detectable mutations in both formalin-fixed paraffin-embedded (FFPE) tumor tissue and ctDNA, a statistically significant correlation between blood-based TMB and tissue-based TMB was found (p=0.002) (109). Tumors with high microsatellite instability (MSI) can also be detected using ctDNA based assays (110). Previous studies demonstrated that high MSI from ctDNA is associated with a good response to ICI across various cancers (111). Additionally, the detection of somatic mutations in cfDNA modulating tumor-specific immune response might be helpful to identify non-responding patients. However, genomic analysis to detect mutations and TMB in blood could contain some mutations associated with clonal hematopoiesis, so these non-tumor mutations should be filtered out to prevent misleading results (112).

Studies on ctDNA in breast cancer patients receiving immunotherapy are summarized in **Table 3**. INSPIRE, a multicohort phase 2 trial, was conducted to evaluate the performance of an amplicon-based bespoke (personalized) ctDNA detection to predict response in patients treated with pembrolizumab (113). This study aimed to investigate if baseline ctDNA levels would be prognostic and whether early changes in ctDNA levels would precede imaging response to an ICI. Five cohorts of patients with advanced solid tumors were included. A total of 106 patients were enrolled; of them, 18 patients were TNBC. Researchers analyzed ctDNA levels at baseline and the beginning of cycle 3 of pembrolizumab treatment. Patients who had a lower ctDNA level at cycle 3 than at baseline, had a higher clinical benefit rate (CBR) and a more favorable OS and PFS. They monitored dynamic levels of ctDNA during pembrolizumab treatment to evaluate the predictive value of ctDNA. Among patients with at least two ctDNA measurements, any rise in ctDNA levels above baseline (n=45) during surveillance was associated with rapid disease progression in most patients and with poor survival (median OS=13.7 months). Patients whose ctDNA cleared during treatment (undetectable for at least one on-treatment time point) had superior clinical outcomes. This study showed that serial ctDNA analysis using the bespoke assay could be a monitoring strategy for patients treated with ICI. Changes in ctDNA levels and Response Evaluation Criteria in Solid Tumors (RECIST) from baseline to cycle 3 were discordant in 23% of cases, but the combination of these two metrics was superior to RECIST alone for predicting OS. This study suggests broad clinical utility for ctDNA based surveillance in patients treated with ICI (113). This is a noninvasive strategy to predict clinical benefit and long-term survival. Future large interventional studies are needed to confirm these results using ctDNA levels to guide ICI therapy.

**Table 3:** Studies on ctDNA in breast cancer patients receiving immune-therapy.

Immunotherapy agent	Setting	Liquid Biopsy Technology	Endpoints	Sample	Results	Reference
Pembrolizumab	Metastatic		PFS, OS, CBR The change in genomics and immune landscapes, RNA expression correlates of treatment response.	316 serial plasma samples Total pts= 94 TNBC=11	Patients who had lower ctDNA level at cycle 3 than ctDNA level at baseline has higher CBR, favorable OS and favorable PFS. Patients whose ctDNA cleared during treatment had superior clinical outcomes.	(113)
Investigational Immunotherapy (ICI, vaccines, cytokines)	Metastatic	Next generation sequencing of a customized panel of genes	To evaluate ctDNA dynamics in responders.	Total=38 BC=5	Blood-based TMB correlated with tissue-based TMB High TMB was not associated with better survival An on-treatment decrease in VAF of mutations detected in ctDNA at baseline was observed in responders.	(109)
Pembrolizumab	Neoadjuvant	Personalized ctDNA test (Signatera™)	Association of ctDNA with with pCR and DRFS	511 serial samples from 138 patients (pembrolizumab arm n=2) HR+/HER2 negative=77 TNB=61	Early clearance of ctDNA during NAC treatment was significantly associated with increased likelihood of achieving pCR Residual ctDNA after neoadjuvant treatment was a significant predictor of metastatic recurrence and death.	(114)

BC, breast cancer; ctDNA, Circulating tumor DNA; DRFS, distant recurrence-free survival; pCR, pathologic complete response rate; pts, patients; TMB, tumor mutational burden; TNBC, triple negative breast cancer; VAF, somatic variant allele frequency.

In early-stage breast cancer, the addition of pembrolizumab to standard neoadjuvant chemotherapy improved pCR rates in patients with HR+, HER2 negative breast cancer and TNBC in the I-SPY2 trial (66). ctDNA levels were analyzed on 511 serial plasma samples during neoadjuvant treatment. The detection of ctDNA decreased over time in both the pembrolizumab arm and the control arm. All patients who achieved pCR (n=34) cleared their ctDNA prior to surgery. Among patients who failed to achieve pCR, the distant recurrence free survival (DRFS) rate was significantly better in patients who had ctDNA clearance prior to surgery compared to patients who were ctDNA positive (114).

## 6 Promises, Pitfalls, and Challenges of CTCs and ctDNA as Biomarkers for Breast Cancer Immunotherapy

CTCs are extremely rare, with an estimated frequency of 1 CTC per one billion blood cells and are difficult to detect in circulation. Counting (enumeration) them requires special reagents (e.g., immunomagnetic beads) and equipment (e.g., automated fluorescent microscope). Current CTC detection technologies, such as that of the CellSearch™ system, have limited sensitivity. Given that CTCs are relatively more abundant in blood of metastatic breast cancer patients, the analysis of CTCs may be more robust in the metastatic setting than in early-stage breast cancer. Even so, only about 50% of metastatic breast cancer patients are positive for CTC (5, 36).

The detection of ctDNA, on the other hand, is less technically challenging than that of CTCs. The isolation of cfDNA, which serves as the input material for sequencing, can be easily performed using commercially available purification kits. The downstream analysis to detect ctDNA in cfDNA generally requires only a next generation sequencer, instrumentation that is available in academic research settings and fee-for-service commercial sequencing companies or clinical reference labs.

Because CTCs can be isolated as live cells, other substrates for biomarker detection and discovery (e.g., DNA, RNA, proteins, and other macromolecules) are available for interrogation. This is a significant advantage of CTCs over ctDNA, which is limited to DNA-based profiling due to the nature of the biomarker (**Table 4**).

**Table 4:** Feasibility of assessment of candidate immunotherapy biomarkers in circulating tumor cells (CTC) and circulating tumor DNA (ctDNA).

Biomarker	CTC	Reference	ctDNA	Reference
DNA-based biomarker	TMB can be measured by DNA sequencing of single or small pools of CTC	(115)	TMB can be measured in cfDNA using a targeted panel or by whole exome sequencing of cfDNA.	(116–120)
			Genome-wide tumor-specific copy-number alterations can be profiled from cfDNA to monitor response to immunotherapy.	(121)
RNA-based biomarkers	Profiling of gene expression signatures associated with immunotherapy response in CTC is feasible.	(122)	n.a.	
Protein-based markers	PD-L1 expression can be assessed by staining of isolated CTC.	(120, 122–124)	n.a.	

cfDNA, cell-free DNA; CTC, circulating tumor cells; n.a., not applicable; PD-L1, Programmed death-ligand 1; TMB, tumor mutational burden.

## 7 Emerging Liquid Biopsy Technologies

In addition to CTCs and ctDNA, other blood-based biomarkers have been recently developed (125, 126). In this review, we will focus on emerging cfDNA-based biomarkers beyond mutation profiling.

Other cfDNA-based biomarkers, in addition to the detection of tumor mutant DNA molecules (i.e., ctDNA) are being developed. Cristiano and colleagues described an approach to profile genome-wide fragmentation patterns of cfDNA, also referred to as “fragmentomics” (127). The authors showed that fragmentation profiling, combined with mutation-based analysis, can accurately discriminate between cancer patients and healthy individuals. Another approach involves methylation sequencing of cfDNA (128). For example, Liu and colleagues showed that evaluation of the methylation patterns in more than 900 CpG sites in cfDNA detected the presence of cancer and identified the cancer type in patients with advanced cancers. Chromatin state or nucleosome footprint analysis of the cfDNA is another approach that is currently under development (129, 130). The positions of nucleosomes on DNA determine chromatin structure which in turn affect gene expression (131). This approach involves generating genome-wide maps that show nucleosome occupancy and the evaluation of transcription factor binding in small fragments of cfDNA (129). Using this approach, Ulz and colleagues found patient- and tumor-specific nucleosome occupancy patterns and were able to accurately predict subtypes in prostate cancer (130).

Mutation detection in cfDNA is challenging because rare tumor-derived mutated DNA molecules are present in an overwhelming background of normal DNA from hematopoietic cells. Detection is particularly challenging in cancers with low or moderate tumor mutational burden, like breast cancer (132). These new emerging platforms offer the opportunity to interrogate genome-wide or significantly more genomic loci than what is available for mutational profiling. For example, Jensen and colleagues describe the use of a genome-wide measure of genomic instability by low-coverage next generation sequencing of cfDNA, an assay that is validated for noninvasive prenatal testing, to detect tumor-specific copy number aberrations (13, 121, 133). Using this approach, the investigators developed a novel metric, genome instability number (GIN), that can be used to monitor response to immunotherapy drugs, including the differentiation of progression from pseudoprogression (121). The GIN assay and other novel technologies that interrogate the whole genome show promise in providing clinically relevant information above what ctDNA alone can provide. However, further testing to demonstrate their applications to guide immunotherapy, particularly in breast cancer, is warranted.

## 8 Future Directions and Summary

Immunotherapy has a defined role in the treatment of both early- and late-stage TNBC and is under active exploration in HER2+ as well as high-risk HR+ disease. Only a minority of patients in the metastatic setting are likely to benefit from adding ICI to standard chemotherapy, and outcome is particularly poor for patients with PD-L1-negative disease. In the early-stage setting, therapy is given with curative intent, so the balance of toxicity and efficacy is critical. In addition, ICI therapy is costly, and the duration of therapy has implications for both toxicity and patient quality of life. It is therefore of the utmost importance to identify better markers to predict efficacy. The analysis of PD-L1 expression on CTCs and the detection of ctDNA are actively under investigation. Confirming the predictive value of TMB in prospective trials and standardizing the assessment of TMB are critical next steps.

Further clinical studies are warranted to demonstrate the role of liquid biopsy in guiding immunotherapy in breast cancer. Blood biomarkers can monitor disease trajectory during and after therapy and have the potential to reveal mutational shifts and resistance mechanisms. These biomarkers reflect, in part, the changes in tumor burden during treatment. However, the correlation between tumor burden/response and the levels of CTCs and ctDNA is not perfect; therefore, additional biomarkers are needed to refine their predictive and prognostic value. Ongoing clinical trials involving the assessment of liquid biopsy technologies in patients with breast cancer receiving immunotherapy are listed in **Table 5**.

**Table 5:** Overview of ongoing clinical trials of liquid biopsy techniques in breast cancer undergoing immunotherapy.

Clinical Trial Number	Setting	#Patients	Assessments	Aim of Liquid Biopsy Analysis	Estimated Primary Completion Date
NCT03892096	Metastatic BC, NSCLC, CRC	750	ctDNA	The evaluation of ctDNA as a potential biomarker for early non-response to therapy	2022
NCT04591431	BC, GIC, NSCLC, other	384	ctDNA	Concordance between molecular profile on tumor tissue and ctDNA	2024
NCT02971761	Metastatic TNBC	29	ctDNA, CTC	To evaluate die effect of the combination therapy (Enobosarm and Pembrolizumab) on CTC and ctDNA.	2021
NCT04849364	Post-neoadjuvant residual TNBC	197	ctDNA	Patients wim residual TNBC assign to arms based on ctDNA positivity and genomic markers).	2024
NCT04837209	Metastatic TNBC	32	ctDNA	To evaluate changes in ctDNA in patients receiving the combination of niraparib, dostarlimab, and RT	2023
NCT04447651	Metastatic BC	60	ctDNA	To evaluate changes in ptDNA from baseline to 3 months in patients with spliceosome mutations receiving ICI	2022
NCT03515798	Inflammatory BC	81	CTC, ctDNA	To evaluate prognostic value of baseline CTC in IBC To purify ctDNA for disease monitoring	2025
NCT03145961	Early-stage TNBC	208	ctDNA	To assess whether ctDNA screening can be used to detect residual disease following standard primary treatment for TNBC To assess the safety and activity of pembrolizumab in patients widi positive ctDNA	2022
NCT03213041	HER2 negative metastatic BC	100	CTC, ctDNA	To evaluate the efficacy of carboplatin+ pembrolizumab in patients with CTC+ metastatic BC To measure ctDNA and correlate them with CTC enumeration and therapeutic benefit.	2022
NCT03818685	TNBC with residual disease	114	ctDNA	ctDNA detection at baseline and in case of disease relapse up to 2 years	2021
NCT03487666	TNBC with residual disease	45	ctDNA	Quantification of ctDNA at different time points during Nivolumab or capecitabine or combination therapy as adjuvant therapy for TNBC with residual disease following neoadjuvant chemotherapy	2021

BC, breast cancer; CRC, colorectal cancer; ctDNA, Circulating tumor DNA; GIC, gastrointestinal cancer; IBC, inflammatory breast cancer; ICI, immune checkpoint inhibitors; NSCLC, non-small cell lung cancer; PC, pancreas cancer; RT, Radiation Therapy. Ongoing clinical trials were found at the website of https://www.Clinicaltrials.gov (accessed on 1 September 2021).

In conclusion, liquid biopsy applications to guide immunotherapy in breast cancer have not yet been implemented in clinical practice, but promising data and rapidly advancing technologies indicate that this approach has the potential to select patients who would benefit from immunotherapy.

## Author Contributions

The authors MM and OG contributed to the study conception, data collection and interpretation, and manuscript preparation. HR wrote sections of the manuscript. HR, RK, and LVV reviewed the manuscript. All authors contributed to manuscript revision, read, and approved the submitted version.

## Funding

The study was funded in part by the grants from the NIH (R01 CA255442), the Breast Cancer Research Foundation (BCRF-18-142), and the California Breast Cancer Research Program IDEA award; and a fellowship awarded to MM by the Cancer Cell Mapping Initiative (U54 CA209891).

## Conflict of Interest

HR received research support for clinical trials through the University of California: Pfizer, Merck, Novartis, Lilly, Roche, Odonate, Daiichi, Seattle Genetics, Macrogenics, Sermonix, Boehringer Ingelheim, Polyphor, Astra Zeneca, OBI, Gilead, and Ayala; honoraria: Puma, Mylan, Samsung, and Napo. RK has received research funding from Biological Dynamics, Daiichi Sankyo, Inc., EISAI, Boehringer Ingelheim, Debiopharm, Foundation Medicine, Genentech, Grifols, Guardant, Incyte, Konica Minolta, Medimmune, Merck Serono, Omniseq, Pfizer, Sequenom, Takeda, and TopAlliance; as well as consultant and/or speaker fees and/or advisory board for Actuate Therapeutics, AstraZeneca, Bicara Therapeutics, Biological Dynamics, EISAI, EOM Pharmaceuticals, Iylon, Merck, NeoGenomics, Neomed, Pfizer, Prosperdtx, Roche, TD2/Volastra, Turning Point Therapeutics, X-Biotech; has an equity interest in CureMatch Inc., CureMetrix, and IDbyDNA; serves on the Board of CureMatch and CureMetrix,and is a co-founder of CureMatch. LVV is a co-founder, stockholder and part-time employee of Agendia NV.

The remaining authors declare that the research was conducted in the absence of any commercial or financial relationships that could be construed as a potential conflict of interest.

## Publisher’s Note

All claims expressed in this article are solely those of the authors and do not necessarily represent those of their affiliated organizations, or those of the publisher, the editors and the reviewers. Any product that may be evaluated in this article, or claim that may be made by its manufacturer, is not guaranteed or endorsed by the publisher.
